# Pre-assessment of patients with suspected axial spondyloarthritis combining student-led clinics and telemedicine: a qualitative study

**DOI:** 10.1007/s00296-023-05522-z

**Published:** 2024-01-30

**Authors:** Katharina Boy, Sophie von Rohr, Susann May, Sebastian Kuhn, Georg Schett, Hannah Labinsky, Johannes Knitza, Felix Muehlensiepen

**Affiliations:** 1 Center for Health Services Research, Faculty of Health Sciences Brandenburg, Brandenburg Medical School Theodor Fontane, Seebad 82/83, 15562 Rüdersdorf Bei Berlin, Germany; 2 Department of Internal Medicine 3, Rheumatology and Immunology Friedrich, Alexander University Erlangen-Nürnberg and Universitätsklinikum Erlangen, Erlangen, Germany; 3 Department of Internal Medicine 2, Rheumatology/Clinical Immunology, University Hospital Würzburg, Würzburg, Germany; 4 Institute for Digital Medicine, University Hospital of Giessen and Marburg, Philipps University Marburg, Marburg, Germany; 5 AGEIS, Université Grenoble Alpes, Grenoble, France

**Keywords:** Telemedicine, Rheumatology, Diagnosis, Diagnostic delay, eHealth, Self-sampling, Symptom checkers

## Abstract

**Objective:**

Patients referred to rheumatologists are currently facing months of inefficient waiting time due to the increasing demand and rising workforce shortage. We piloted a pre-assessment of patients with suspected axial spondyloarthritis (axSpA) combining student-led clinics and telemedicine (symptom assessment, symptom monitoring and at-home capillary self-sampling) to improve access to rheumatology care. The aim of this study was to explore (1) current challenges accessing axSpA care and (2) patients’ first-hand experiences.

**Methods:**

Embedded within a clinical trial, this study was based on qualitative interviews with patients with suspected axSpA (*n* = 20). Data was analysed via qualitative content analysis.

**Results:**

Student-led clinics were perceived as high-quality care, comparable to conventional rheumatologist-led visits. Patients expressed that their interactions with the students instilled a sense of trust. History-taking and examinations were perceived as comprehensive and meticulous. Telehealth tools were seen as empowering, offering immediate and continuous access to symptom assessment at home. Patients reported a lack of specificity of the electronic questionnaires, impeding accurate responses. Patients requested a comments area to supplement questionnaire responses. Some patients reported receiving help to complete the blood collection.

**Conclusion:**

Patients’ access to rheumatology care is becoming increasingly burdensome. Pre-assessment including student-led clinics and telemedicine was highly accepted by patients. Patient interviews provided valuable in-depth feedback to improve the piloted patient pathway.

**Supplementary Information:**

The online version contains supplementary material available at 10.1007/s00296-023-05522-z.

## Introduction

Axial spondyloarthritis (axSpA) is a rheumatic disease that predominantly affects the spine and may be associated with peripheral joint disease and extra-articular organ manifestations. The prevalence of axSpA ranges from 0.4 to 2% [1]. Initial symptoms, especially chronic lower back pain, are often misinterpreted, leading to delayed diagnosis and treatment. The growing supply–demand mismatch in the rheumatology workforce contributes to a significant further delay, which for axSpA patients is approximately 7 years [2–4]. Untreated disease is associated with a worsening prognosis and quality of life and causes functional limitations [5]. Several strategies, such as delegation of tasks and implementation of telemedicine have been proposed to compensate for the workforce shortage and resulting negative impact on clinical care, such as diagnostic delay [6]. Delegation of tasks could reduce the workload of rheumatologists. Traditionally, nurse practitioners and physician assistants aid rheumatologists seeing new patients [7]. Early clinical exposure for medical students is widely encouraged and appreciated by the majority of students. However, when compared to other fields such as diabetology, actual integration of medical students into clinical routine with documented positive effects is scarce in rheumatology [8, 9]. Accelerated by the COVID pandemic, rheumatology has experienced a major uptake of telemedicine [10]. The evidence regarding the implementation of telemedicine in rheumatology remains however scarce and more evidence [11] is desperately needed [10]. To guide clinicians the European Alliance of Associations for Rheumatology (EULAR) published the first official points to consider for remote care in rheumatic diseases in 2022. Major recommendations were to use telehealth for a pre-assessment to improve the referral process and to monitor symptoms and disease activity. In previous studies we could demonstrate that telehealth tools such as symptom checkers, monitoring apps and capillary self-sampling are appreciated by rheumatic patients [12–14].

In a pilot study we combined these telehealth tools with student-led clinics to enable a pre-assessment of patients with suspected axSpA. The use of telehealth tools and the pre-assessment by students while waiting for the regular appointment with a rheumatologist could accelerate axSpA diagnosis and therapy [8].

The aims of this embedded qualitative study were to explore (i) current challenges accessing care and (ii) patients’ first-hand experiences with the piloted pre-assessment patient pathway, including acceptance, perceived benefits and drawbacks.

## Methods

The study was approved by the institutional review board (IRB) of the Medical Faculty of the University of Erlangen-Nürnberg, Germany (21-357-B) and conducted in compliance with the Declaration of Helsinki. All patients provided written informed consent prior to study participation. Consecutive, newly referred patients with chronic low back pain for at least 3 months from the outpatient clinics of the Department of Rheumatology at the University Hospital Erlangen were included. Further inclusion criteria were a minimum age of 18 years, sufficient language skills, and regular usage of a smartphone. Exclusion criteria were an established rheumatic diagnosis and unwillingness or inability to comply with the protocol.

**Fig. 1:**
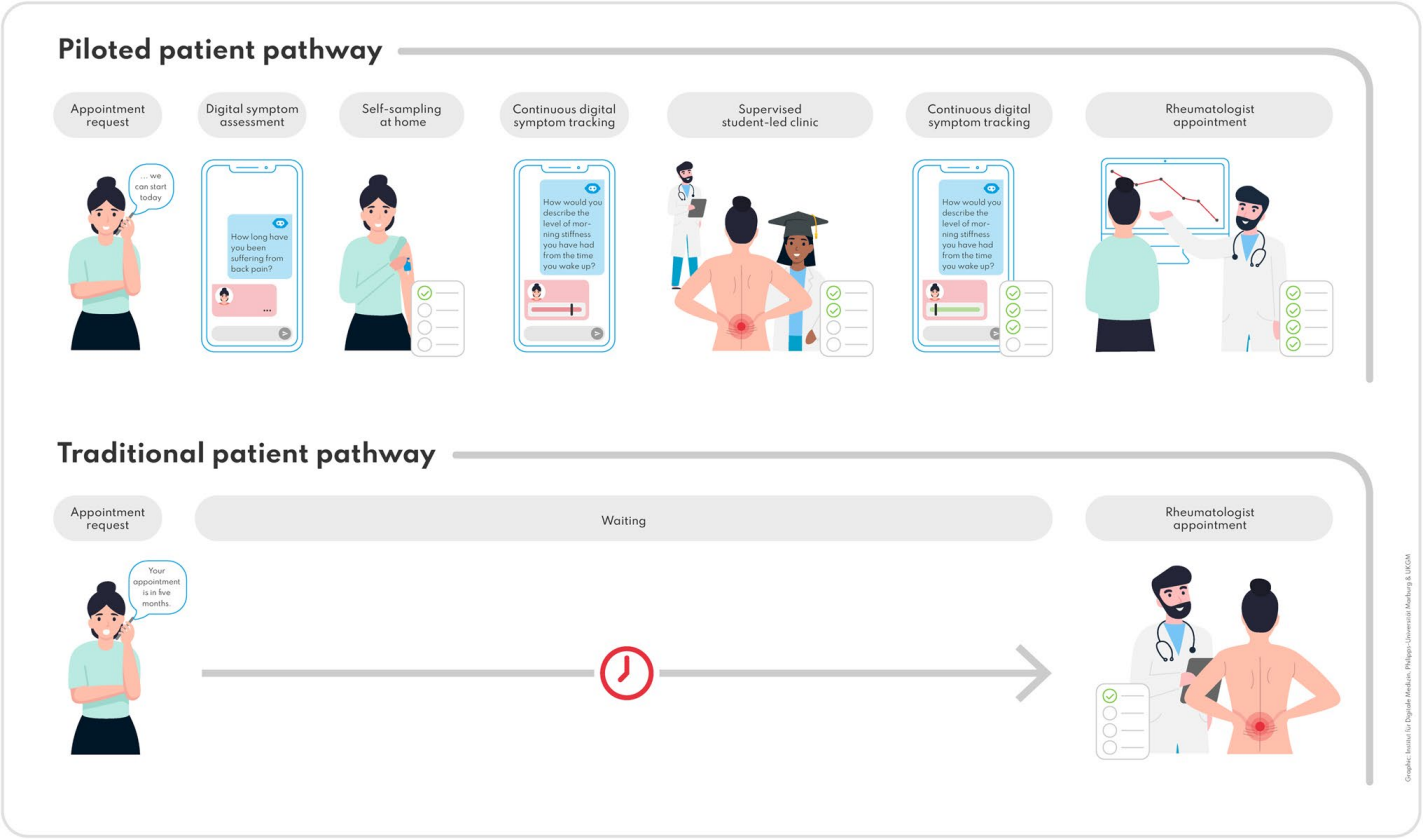
Overview of the piloted patient pathway and traditional patient pathway

The piloted patient pathway and traditional patient pathway are depicted in Fig. 1. The methodology has been presented in more detail in previous publications [8, 15, 16].

The traditional patient pathway (bottom) and the piloted patient pathway (top) are shown. Between the appointment request and the regularly scheduled rheumatology appointment, study patients were offered digital symptom assessment with a medical app and at-home blood self-sampling, as well as a supervised student-led clinic to progress the diagnostic workflow in the waiting period.

Briefly, patients with suspected axSpA waiting for their rheumatology appointment were offered telehealth tools (two symptom checkers, a medical app to monitor disease activity, at-home blood-testing of HLA-B27 and CRP) and student-led clinics prior to the routine rheumatology appointment. The fourth year medical student independently studied axSpA disease and shadowed rheumatology residents to prepare a standardized patient evaluation.

To explore the patients’ experiences with the new care model, we conducted qualitative interviews. To reduce the risk of infection and to reduce the burden on patients, interviews were conducted by telephone. The interviews took place between March and September 2022. 20 participants were selected using purposive sampling [17] to include a heterogeneous sample in regard of age, sex, educational and professional background of the patients interviewed. Participants did not receive financial incentives. The interviews were conducted using an interview guide that was developed to specifically elicit the participants’ experiences. The semi-structured interview guide (Supplemental Material) consisted of open-ended questions that explored the user perspectives towards the new care model. The following main topics were investigated: acceptance, benefits and drawbacks, and transferability to standard care. The initial exploratory questions were then refined through follow-up questions. We conducted three pilot interviews to test and refine the interview guide. No revisions were necessary. In addition, socio-demographic data was collected, including gender, age, diagnosis, education and occupation. Data collection and analysis was conducted by a MD student (KB) and two health services researchers (SM and FM) based on Kuckartz’s structured qualitative content analysis [15] using MAXQDA software (Verbi GmbH). After transcription of the audio material, the analysis began with a familiarization with the interviews, whereupon the interviews were coded (KB, SM, FM). The categories were developed inductively to capture the relevant material in the transcripts using the data-driven development of a coding tree (Supplemental Material).

Subsequently, the category system was applied to the entire qualitative data. At this point, the data collection had already been completed. Representative quotes from the transcripts were selected, translated into English and included in the manuscript to present the results. The manuscript has been compiled in accordance with the Consolidated Criteria for Reporting Qualitative Research (COREQ) (Supplemental Material) [18].

## Results

### Patients characteristics

Mean age of interviewed patients was 44 (range: 22–65) years, see Table 1. 13/20 (65%) of patients were female. Patients reported diverse occupational and educational backgrounds. All patients had a suspected axSpA diagnosis. The interviews lasted between 8 and 33 min (mean 15.3, 25).

**Table 1:** Participant characteristics

Patient	Age (years)	Gender	Education	Occupation	axSpA
1	51	Male	Vocational baccalaureate diploma	Office worker electrical engineering	Yes
2	43	Male	vocational baccalaureate diploma	Subway driver	Yes
3	65	Female	Secondary school diploma	Office clerk	Yes
4	50	Female	High School degree	Nurse	No
5	51	Female	High School degree	Nurse	Yes
6	55	Female	High School degree	Administrative employee	No
7	56	Female	High School degree	Administrative employee	No
8	57	Female	High School degree	Administrative employee	Yes
9	35	Female	Secondary school diploma	Office clerk	Yes
10	51	Female	Secondary school diploma	Salesperson	Yes
11	45	Male	Secondary school diploma	Landscape gardener	Yes
12	45	Female	University degree	Social pedagogue	Yes
13	51	Female	University degree	Product manager	No
14	28	Male	Secondary school diploma	Car mechanic	Yes
15	35	Male	University degree	Engineer	Yes
16	22	Male	Secondary school diploma	Landscape gardener	No
17	53	Female	Secondary school diploma	Office employee	No
18	32	Female	Secondary school diploma	Paralegal	No
19	32	Male	Secondary school diploma	Construction worker	Yes
20	34	Female	Secondary school diploma	Graphic designer	Yes

### Current challenges with traditional patient pathway

Long travelling distances and long waiting times at the clinic were described as burdensome and challenging to integrate into personal daily life.

*“For me it is sixty kilometers. So, depending on where you have to go, it is of course a hassle.” (P 8, pos. 35)*

*“I mean, I have children, I’m employed. That’s already an enormous time expenditure, currently also with the search for a parking space. Then somehow registering again. And waiting times on site.” (P 20, pos. 37)*

Patients pointed out to suffer for a long time and to experience high levels of psychological strain. Insecurities were often described, also due to bad previous medical experiences.

*“So, you know, if you've been walking around with pain for five years or even longer, you're just glad for once that someone is there to take care of you. And also takes you seriously. Because these are diseases where you are not taken one hundred percent seriously by many doctors.” (P 17, pos. 39)*

Patients reported that doctors have limited time, which might affect their quality of work and lead to medical errors.

*“Everyone knows that when you're at the doctor's, everything always has to be done quickly. The doctors always don't have time, and they only listen to half of what you say. It's like that everywhere, no matter where you are.”(P 5, pos. 53)*

The patients highlighted the new model of care as an option to reduce waiting times for their rheumatology appointment.

*“That was actually quite good, because it meant I got an appointment relatively early. If I had registered normally, I think I would have had to wait a minimum of three months for an appointment.” (P 18, pos. 41–43)*

### Experiences with piloted pre-assessment-based patient pathway

Patients described long waiting times to receive their rheumatology appointment in the traditional patient pathway and welcomed the new care model offering faster appointments.

Patients were able to give a comprehensive description of the piloted patient pathway, including precise descriptions of all key components. As the interview progressed, each component was addressed, and the patients' perspectives were elicited.

#### Student-led clinics

Patients experienced the student-led clinics as high-quality and non-inferior to standard care. Participants commented more extensively on their experiences with the student than on the other components of the pre-assessment. Patients perceived the student-led clinics as an effective preceding supplement to the rheumatologist appointment. Patients described their experiences, which are shown in Table 2.

**Table 2:** Student-led clinics patient experiences

Category	Anchor quote
High-quality and non-inferior to standard care	*“So I was told beforehand that there were two appointments and I thought it was good, because otherwise the waiting time would have been relatively long. The student appointment shortened that considerably, I don't remember exactly, but it was definitely much earlier and finally also shortened the actual doctor's appointment quite a bit. Yes, it was good.” (…) So you went straight in and didn't have to do another hour of anamnesis.” (P 15, pos. 23, 31–33)* *“So from start to finish, that was super, really top notch. I never had the feeling that the students didn't care, or were heartless or anything, no. Top. One hundred points, one hundred percent. I can't say anything negative about that, absolutely not.” (P 11, pos. 30)*
Awareness of workforce shortage and delegation to students	*“It is okay that a doctor, who certainly also costs more, does not necessarily have to do the initial examination. I think a student can do that first, asking questions about how the disease has been perceived so far, what symptoms are there or not. So, I find that really good.” (P 2, pos. 35)*
Competent, interested, sometimes even superior to physician-provided care	*“So, if that had been a blind test now, I probably wouldn't have recognized any difference from a doctor.” (P 15, item 21)* *“I often work with students professionally and can say they are often more thorough than the registered doctors. My experience is that students/ inexperienced doctors often check everything ten times, try to take every possibility into account, and that doctors with more experience are quick to say: "Oh, no, that and that, of course.” (P 4, pos. 37–39)*
Benefits for rheumatologists and students	*“The student is not only there and observes, but also registers patients himself. So, writing, listening, talking, participating. One learns much more with it, than if one only runs along as a student.“ (P 13, pos. 21)* *“Yes, so I definitely assume that it is simply an advantage for the doctor because all these preliminary discussions have already taken place, because he simply has a basis on which he can continue.” (P 10, pos. 27)*

Patients could very well imagine student-led clinics as part of standard care.

*“Yes, I can well imagine that in the future. As soon as it is standardized and also discussed afterwards with the doctor and perhaps prepared in advance so that the important questions and information are considered.” (P 12, pos. 45)*

Patients also reported limitations. Information regarding treatment options were only addressed together with a rheumatologist.

*“Well, okay, if you now assume that it's a patient like me, who has many, many questions, a student can't answer them, of course, so you have to wait until you actually go to the doctor.” (P 10, pos. 37)*

Patients provided various suggestions to improve student-led clinics displayed in Table 3.

**Table 3:** Patient suggestions to improve student-led clinics

Category	Anchor quote
Written document	*“If I had a printout with the questions, that could be used in a long-term way. Because if you have any chronic diseases or diseases that are composed of different specialties: I was at the orthopedist, at the family doctor and internist. And you have to tell the same story every time, over and over again. But then I would also have something where I say: okay, I'll copy that.”* *(P 13, pos. 41)* *“Just, as I said - you just go home and have no written document.”* *(P 5, pos. 67)* *“So, I would be happy about such a preliminary letter or so, that the student writes something preliminary. I fully understand that I won't have the appointment until a later date. Just in case, so I would have something in writing.” (P 6, pos. 111)*
Therapy advice	*“If I have pain again, what should I do? I've had massages now, and actually they didn't do me any good. Should I continue? Should I go to the massage despite the pain? Or should I stop? Just tips. Or which medication would be better, or not so good? Or just, yes, also other things, what could help, ne?” (P 5, pos. 61)*
Contact person, accessibility, communication	*“And that would also be a way of communication - that doesn't mean that I want to write an e-mail to this doctor, this nurse or student, whatever, every day. But I would like to have the opportunity, if something is wrong or need to.” (P 13, Pos. 37)*
Shorter time interval between student-led clinic and doctor’s appointment	*“The problem is now, the interval is a little big between the first and the next appointment. So from December to June is a little long, half a year. “ (P 3, pos. 37)* *“I think between this first appointment and the following one (pre-scheduled rheumatology appointment), maybe there shouldn't be so much time. So I wouldn't mind if these appointments were timed a bit tighter. Because then you are hanging there, again for six or eight weeks. (…) Where, if you have a crisis, you just reach your limits.”* *(P 17, pos. 55–59)*

#### Symptom checkers

Overall, patients described the use of symptom checkers as a good way to prepare for the appointment and also to effectively bridge the waiting time.

*“And you could also integrate that into the waiting time at the beginning. (…). It could take away some people's anxiety or help if one lacks the words or so.” (P 13, pos. 59)*

The results of the symptom checkers provide initial information about the illness and enable patients to work on resources and exchange information with others during the initial diagnosis.

*“And so you already have a rough idea of what it could be. I talked to a good friend of mine and with his mother. They both have rheumatism. And they could already tell me something about it from their experiences.” (P 14, pos. 103–107)*

The personalized questions, simple handling, as well as ecological aspects (digital processing) were positively assessed.

*“So, I think it's helpful. And it also replaces this complete paperwork, jumble, which is always built up like this - Paper, paper, paper, paper.” (P 7, pos. 55)*

Some patients indicated that using symptom checkers has the potential to cause uncertainties and confusion among patients in regard to their diagnosis and symptoms.

*“So if you enter your data as a layman without professional support resulting in various potential diseases, that's just like when you enter any symptoms on Google. I think this is rather counterproductive without having a doctor next to you. Well, you hang a bit in the air and you start thinking, well, do I have that or is it that or not or? So that's actually rather unsettling and confusing, I think.” (P 18, pos. 73–79)*

In addition, some patients experienced difficulties to complete the symptom checkers:

*“So often it's two, three things that you could tick. Sometimes what you are missing or how you experience something is not easy to describe, especially if you can only tick something.” (P 17, Pos. 17)*

#### Disease activity monitoring app

Overall, the patients reported high usability in terms of simplicity, comprehensibility, and flexibility of the app. Above all, the continuous documentation of symptoms over time was positively highlighted. Some patients also perceived the app to be useful to prepare for the next doctor’s appointment.

*“I also found it quite good with the app that you documented this over a long period of time and not always just in a period from doctor's visit to doctor's visit, but also kept track of the time in between.” (P 16, pos. 43)*

*“Well, I definitely use the app. If I have appointments and there are months in between, then of course they want to know how things have improved or deteriorated in the last three or four months. Of course, I don't know anymore, and with this app, I think it's actually quite good.” (P 19, pos. 30)*

Patients appreciated the reminder function of the app and suggested more adaptive reminder intervals. Furthermore, a comment function, as well as a documentation option for medication intake and a help button were mentioned as useful additions.

*“So what bothers me a bit is that the interval is always periodic. So maybe you could make it more intelligent, that it is somehow evaluated, that if I just enter the same thing five weeks in a row, that then somehow the interval is maybe reduced a bit, that it then only comes once a month or so. So that would be, I think, already helpful.” (P 15, pos. 51)*

*“It worked great. Except for two questions, I didn't know exactly (…) who they meant and what time they meant. And then I didn't have a help button where I could somehow get another explanation: okay, in the question this and this and this and this is meant.” (P 13, pos. 55)*

*“But I would sometimes like to be able to set my own settings for such apps and say: okay, I can enter my state of health there every evening if I want to. For example, did I also take painkillers? I would also miss that now. Because, for example, I only take one Ibuprofen at a time, nothing more, nothing at all. But I don't know anymore: when was it so severe that I decided to take an Ibuprofen (NSAID), for example?” (P 13, pos. 67–69)*

Patients did not notice any immediate changes in their rheumatology care as a result of using the app, yet it provided them with a sense of security.

*“So the care is now not yet changed by the app. I do not feel medically cared for by it. I rather feel, which is perhaps more of a point, that I feel somehow in good hands. Definitely! I think to myself: Okay, someone is now (laughs) consciously looking at it. And I am now in good hands. And everything is checked once comprehensively.” (P 20, pos. 25)*

#### Capillary self-sampling

Most patients described the independent collection of capillary blood as quick, easy and painless. The instructions were perceived as clear. Potential savings of travel and time were most frequently mentioned in terms of benefits. Furthermore, it was mentioned that patients do not need a referral under these conditions and that many services related to blood collection are also eliminated (staff, storage, pick-up service to the laboratory). Interviewees also perceived capillary blood collection at home as easy and enjoyed the optimal conditions at home (quiet, familiar environment).

*“So it's awesome in terms of time, because it doesn't take a lot of time. As I said, you put on the cuff, take the device, hit it, poof, fill the cannula, off to the box, off to the post office and goodbye. Brilliant. That's absolutely brilliant. Well, that's what I would wish for, that it comes onto the market, because it's absolutely a super highlight, a super gadget, really. I think that's really awesome. So, someone really thought about it.” (P 11, pos. 54–56)*

Other patients described challenges that they were personally unable to do the capillary self-sampling on their own, so they took the kit to the family doctor or had relatives help them.

*“I was a little nervous, I must admit. But as I said, and then really amazed, because it was so easy.” (P 12, pos. 23)*

While other patients described challenges.

*“I didn't make it. That's why I went to my family doctor and he did it. And the doctor's assistant also said that we couldn't do it alone and I don't know, maybe that was somehow difficult for us.” (P 9, pos. 73–77)*

*“It's just funny, a funny situation. Well, we did it together, my husband and I, he started and pressed the button. But if I didn't have a husband, I would have had to do it alone, right? It's just simple, the diabetics, they have to inject themselves regularly, they're more used to it. But for me it was very unusual. I didn't like that very much. Now in retrospect I have to laugh.” (P 6, pos. 97)*

Some patients reported to end up with a scar after using the self-sampling device.

*“The only thing I notice is that when you take a blood sample using the syringe blood, there is only a fine sting. You get a plaster on it and you don't even see it anymore. Now with the device, you're left with this kind of round ring, the size of a penny piece, it's pricked over such a large area. Or if you do it very often, then in principle you have rings like that, circles like that on your arm. (laughs) It's like a pattern band.” (P 7, pos. 19)*

Furthermore, ecological aspects and costs were addressed critically:

*“I was just thinking what a hassle and how expensive. I mean, I threw away all those materials, right? Well, you had to dispose of them, because they were all single-use materials. That's a bit of a shame, isn't it?” (P 4, pos. 17)*

Patients were grateful for the extended time provided for student clinics.

*“I had the feeling that there was a great time slot for me. The student took a lot of time for me. That has to be said so clearly. She showed me everything, even with the app, and how we did the questionnaires - there was never any time pressure or anything like it. So, that's already different than when a doctor drops by quickly and is gone again right away.” (P 5, pos. 53)*

Patients felt that reducing visits and trips to the rheumatology office saved them time and money. The pre-assessment was easy to integrate in their daily lives.

*“At first, I was sceptical because I have not experienced it that way before. When I go to the doctor, there was always a doctor to talk to. It was the first time for me that I spoke with a student who prepared the whole thing. I also thought that the combination with the app was quite good, that it was documented over a long period of time and not only in a period of time from doctor's visit to doctor's visit, but also the time in between was kept in mind.” (P 16, pos. 42–43)*

*“It saves me a trip to the doctor. It's definitely more helpful that way. Simply in terms of the whole process - for the medical staff and for the patients themselves. For example, I drive 25 kms every time I need to take a blood sample.” (P 17, pos. 79)*

### Potential benefits

**Fig. 2:**
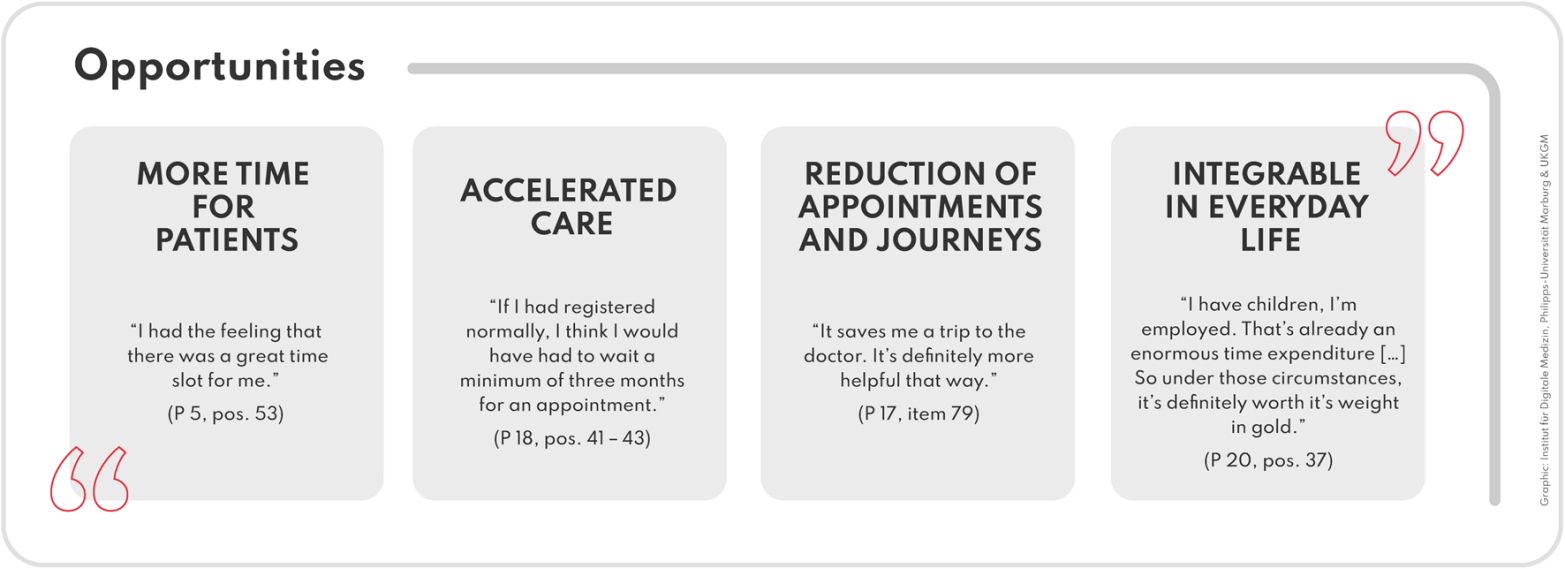
Benefits of the new care model from the patient’s perspective

The transferability of the new care model is reflected in everyday sustainability, time aspects such as reduced waiting time, general travel and time savings. With the student-led consultation hours, an early medical access with a generous time frame is created (Fig. 2).

### Potential barriers

**Fig. 3:**
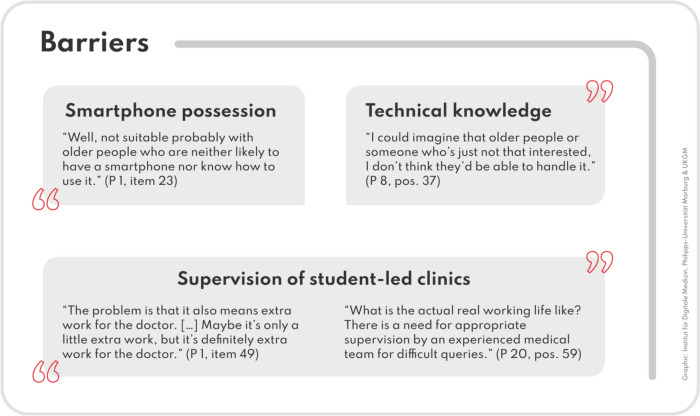
Limitations of the new care model from the patient’s perspective

A number of patients indicated that smartphone possession, as well as technical knowledge, can be a barrier in the context of self-sampling and app usage (Fig. 3).

*“I think that's only something for people who know how to use a smartphone or who are simply technically skilled. I could imagine that older people or someone who's just not that interested, I don't think they'd be able to handle it.” (P 8, pos. 37)*

Patients suggested that the training for students and the effort involved in further teamwork could be a barrier.

*“The problem is that it also means extra work for the doctor. For example - He has to communicate with the student about the patient. Maybe it's only a little extra work, but it's definitely extra work for the doctor.” (P 1, pos. 49)*

## Discussion

In this study, we elucidated experiences of patients with suspected axSpA accessing rheumatology care and completing a pre-assessment-based patient pathway. Pre-assessment was based on student-led clinics and asynchronous telehealth tools, including symptom checkers, a capillary self-sampling device and a medical app to remotely monitor disease activity. To the best of our knowledge, this is the first study to conduct a qualitative assessment of a patient pathway based on delegation of tasks to medical students and telemedicine. Current access to rheumatology care was described as burdensome. Patients confirmed the long diagnostic delays reported in literature [6] and welcomed an accelerated assessment. In line with the quantitative data [8] patients rated the clinical care provided by the student as excellent. Interestingly, some patients even experienced the care as more thorough, pleasant and overall superior to care provided by experienced physicians. Student appointments took roughly one hour and hence were indeed significantly longer compared to average rheumatologist appointments of 15 min. Main suggestions to improve the pre-assessment focused on patients receiving a summary report with treatment recommendations and having the opportunity to ask questions in between appointments. Positive experiences from other disciplines such as dentistry [19], where medical students are carrying out hands-on treatment should further encourage the involvement of medical students in rather conservative disciplines such as rheumatology. The investigated telehealth tools, including symptom checkers, capillary self-sampling, and a monitoring app, were generally welcomed by patients. The high acceptance confirms previous results [8, 12, 14, 20]. The concept of patient empowerment through telemedicine, of being able to actively help collect data for the rheumatologist at home, was well received. In line with previous results [13], patients suggested additional app functions such as a “add note”, “help” and “appointment reminder” function for the monitoring app. Patients also expressed dissatisfaction with having to enter the same data at regular intervals and advised a more flexible monitoring approach. Current evidence supports the idea that patients in remission should be burdened with questionnaires less frequently [16] otherwise, it is likely that app adherence will suffer [21]. Patients also mentioned a lack of technological equipment and understanding as general impediments to telemedicine, which poses the risk that technically inadequately equipped and insufficiently trained patients could be excluded from our model. The mentioned barriers of self-sampling, including production of waste [14] (single-use product) and irreversible scars [22] confirm observations from previous studies. Patients correctly understood that the implementation of such an interdisciplinary care model requires a considerable and continuous team effort, which could be a major barrier for some institutions.

### Limitations

There are some limitations to this study. Participants commented more extensively on their experiences with the student than on the other components of the pre-assessment. The student-led clinic was provided by one individual student (SR). Further validation of the encouraging results with more students and at additional institutions is necessary to prove the cost-effectiveness of the care model. To make student-led clinics sustainable, they should be firmly embedded in the curriculum.

Due to the sampling strategy, we may not have been able to reach everyone and all patient experiences. A generalization of the results is therefore impossible. Furthermore, the perspective of the healthcare providers is not considered in this study. In general physician time was however saved implementing the appreciated pre-assessment approach. Future studies could focus on the perspective and experiences of the students as well as the rheumatologists. Further research could focus on the training of students and the observation and collaboration of doctors to ensure a high quality of healthcare.

## Conclusion

Patients with suspected axSpA perceived the current access to rheumatology as burdensome and welcomed the trialed pre-assessment patient pathway. Implementation of student-led clinics and telemedicine improved access to rheumatology care. Further refinement derived by this study may improve the piloted pathway.

### Supplementary Information

Below is the link to the electronic supplementary material.

Supplementary file1 (DOCX 29 KB)

Supplementary file2 (DOCX 4322 KB)

Supplementary file3 (DOCX 71 KB)

## Data Availability

The datasets used and/or analysed during the current study are available from the corresponding author on reasonable request. All data relevant to the study are included in the article or uploaded as supplementary material. For further questions regarding the reuse of data, please contact the corresponding author (K.B.).
