# Supplementary material for: Pre-assessment of patients with suspected axial spondyloarthritis combining student-led clinics and telemedicine: a qualitative study
Source: Rheumatol Int. 2024 Jan 30;44(4):663–73. doi: 10.1007/s00296-023-05522-z (PMC10914903; doi:10.1007/s00296-023-05522-z)
Supplement: Supplementary file 1 — Supplementary file1 (DOCX 29 KB) [file 296_2023_5522_MOESM1_ESM.docx]

**TELESpActive** **- "Patient:in" guide**

**PATIENT COHORTS 1: A symptom checker, B Student-led clinics, C ABATON, D Capillary self-sampling**

| You have taken part in the following study:  **"Pre-assessment of patients with suspected axial spondyloarthritis combining student-led clinics and telemedicine: a qualitative study.** "  Please describe what you did in the study. | Can you tell us more about this?  And then?  How was that for you?  How do you see that? Can you please go into this in more detail?  Could you please give an example?  What do you mean in concrete terms?  Can you tell us more about this?  And then?  How was that for you?  How do you see that? Can you please go into this in more detail?  Could you please give an example?  What do you mean in concrete terms?  Can you tell us more about this?  And then?  How was that for you?  How do you see that? Can you please go into this in more detail?  Could you please give an example?  What do you mean in concrete terms?  Can you tell us more about this?  And then?  How was that for you?  How do you see that? Can you please go into this in more detail?  Could you please give an example?  What do you mean in concrete terms? |
| --- | --- |
| A Could you please describe your experience with the **symptom checkers**?   - What advantages do you see in the use of digital symptom checkers? (time saving / quality in the appointment) - What disadvantages do you see? - How helpful did you find using the Symptom Checker? - Which patient groups can use Symptom Checker? For whom are they not suitable? |  |
| B Could you please describe your experiences with **Student-led clinics**?   - What was your first thought when you heard about the idea of a student-led clinic? - What motivated you to take part? - What advantages do you see in setting up such a consultation? - What disadvantages do you see? - Why would you recommend / or not recommend the Student Consultation Hour? - What do you think: is the student early consultation hour transferable to standard care? - Do you think it would have made a difference if a doctor had led the consultation? - Is there anything you would improve about the whole procedure? ...or what would you prefer differently? - Do you think an early student consultation before the actual appointment with the doctor could save time or improve the quality of the on-site appointment? - - Is there anything else you would like to address? |  |
| C Could you please describe your experience with the **disease activity monitoring app** (ABATON) ?   - What works well? What doesn't? - How does the app change your rheumatological care? - Could you imagine continuing to use the app?  If yes, under what conditions? If not, why not? |  |
| D Could you please describe your experience with **Capillary self-sampling**?   - If the blood test was commercially available, would you use it?   (to find out about your genetic risk (HLA-B27) and the necessary inflammation levels)? - Would you like to discuss the results with a doctor for categorisation? - What advantages do you see in patients being able to take blood samples themselves at home? (if no suggestions are made e.g. shortening diagnosis, saving appointments etc...) - What disadvantages do you see? |  |
| - Is there anything else you would like to address? - Have you noticed anything else? |  |

| **Age** | **Gender** | **Since when spa diagnosis?** |
| --- | --- | --- |
|  |  |  |
| **Profession** | **Highest educational qualification** | **Notes** |
|  |  |  |
