# Supplementary material for: Pre-assessment of patients with suspected axial spondyloarthritis combining student-led clinics and telemedicine: a qualitative study
Source: Rheumatol Int. 2024 Jan 30;44(4):663–73. doi: 10.1007/s00296-023-05522-z (PMC10914903; doi:10.1007/s00296-023-05522-z)

Stimulating

mindfulness

Stimulation for self-

reflection

Bridging the waiting

time

Symptom checker

Advantages

Notes

Comprehensibility

Symptoms are situational

Promotes uncertainty

Simple handling

Targeted query

Various diseases possible

Focus on symptoms

Time saving

Restricted use

Preparation - appointment

Superficiality

double question

focussed treatment

Ecological aspects

Disease information

Categorisation difficulties

Individual differences

misguided judgement

Lack of complexity

*Resource work & exchange*


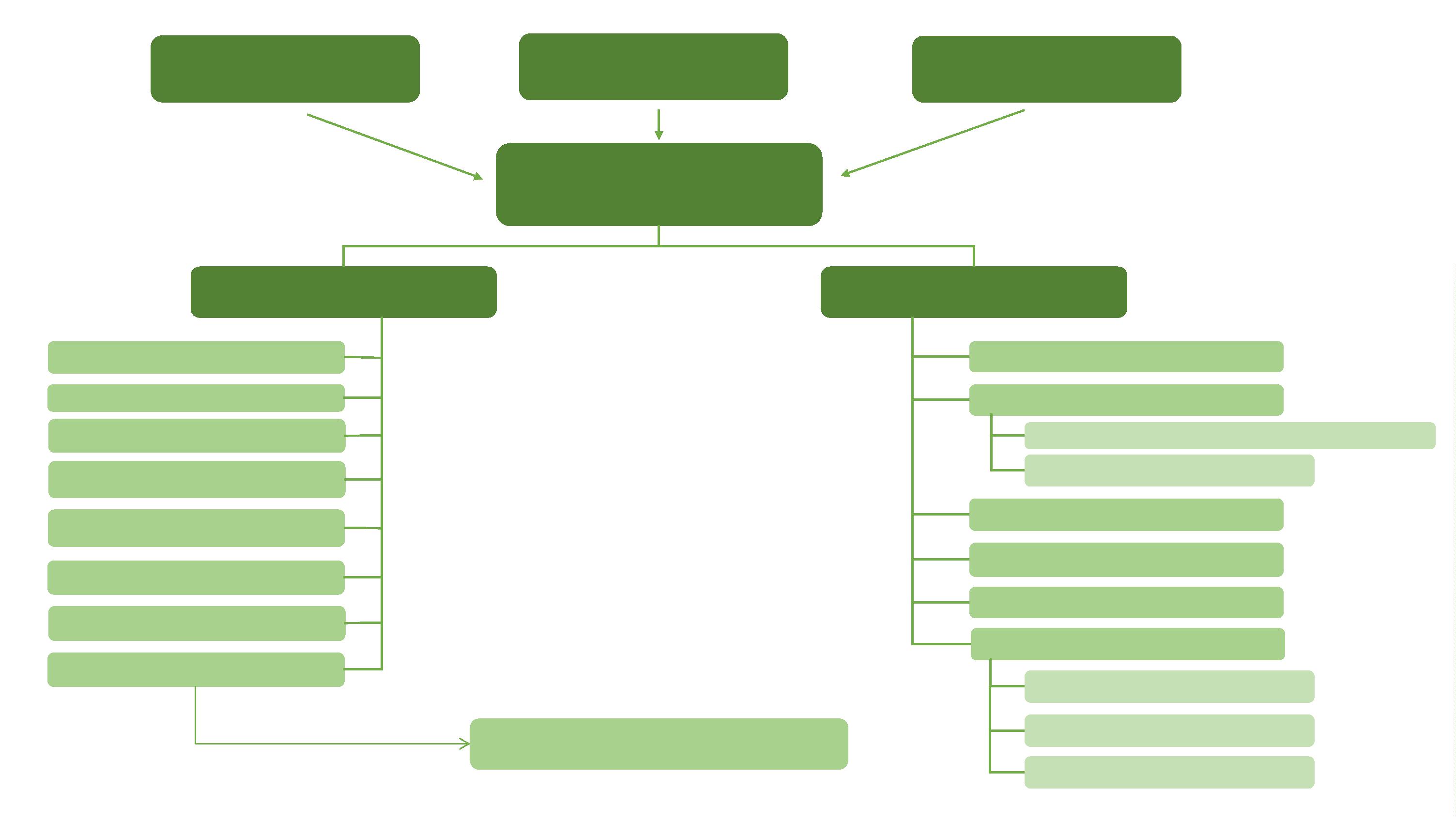


Disease activity

monitoring app

future

Utilisation

Personal user

behaviour

Positive aspects

Notes

Change

the rheumatological

Supply

Further utilisation

options

sensible

regularly

+

Reminder function

trouble-free function

individual questions

Query medication

Flexible

mainly externally

motivated

Customised query

interval

none

analogue

Pain

patients

Diagnosis-related

Diagnosis-related

Study-bound

corresponds to

personal

Feeling cared

for

Help function

Limits

Progress

documentation

preference

Comment function

Termination function

none

Date preparation

Ecological aspects

Handling

not compatible

with everyday

life

Serious

illnesses

Transmission of data

to doctors

Use on different end

devices

technical

Prerequisites

additional

Reminder function

Dates

understand

able

self-

Age

explanatory

Flexible

simple


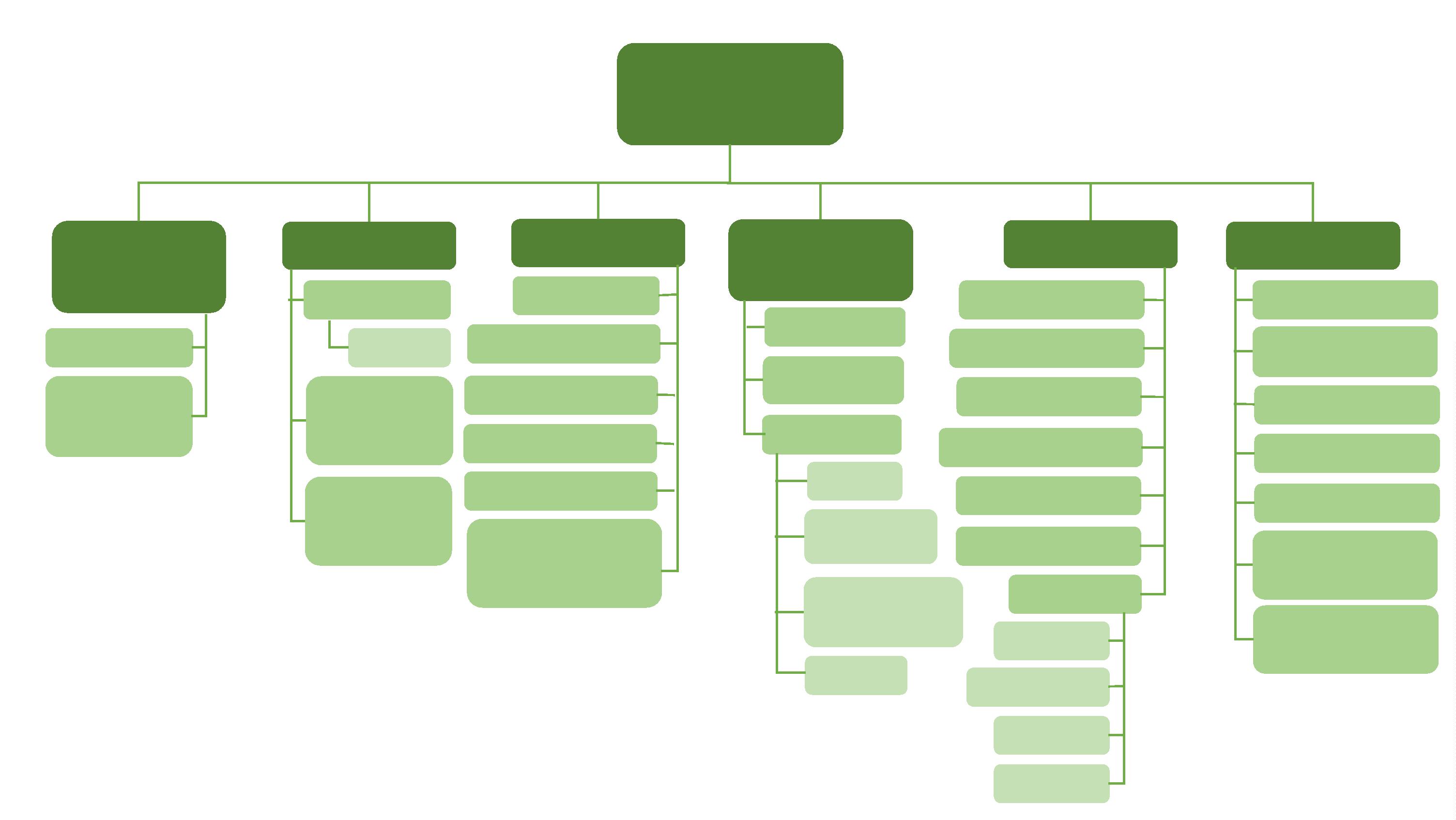


Capillary self-sampling

Uncertainties

Advantages

Disadvantages

Handling

Unfamiliar situation

Progress documentation

suitable for everyday use

Tapping point

Sources of error

Quantity of sample

material

Ecological aspects

(disposable products)

Fear

Assistance

Overcoming

Tapping point

(accessibility)

quiet

Environment/Circumstances

Costs

painless

Comprehensibility

Large tapping point

Time saving

Distance saving

Skin reaction

Restricted use

Savings on bank transfers

Service saving hospital

(Collection of samples)

Age

Skill

Handling

Overcoming

Technical understanding/ interest


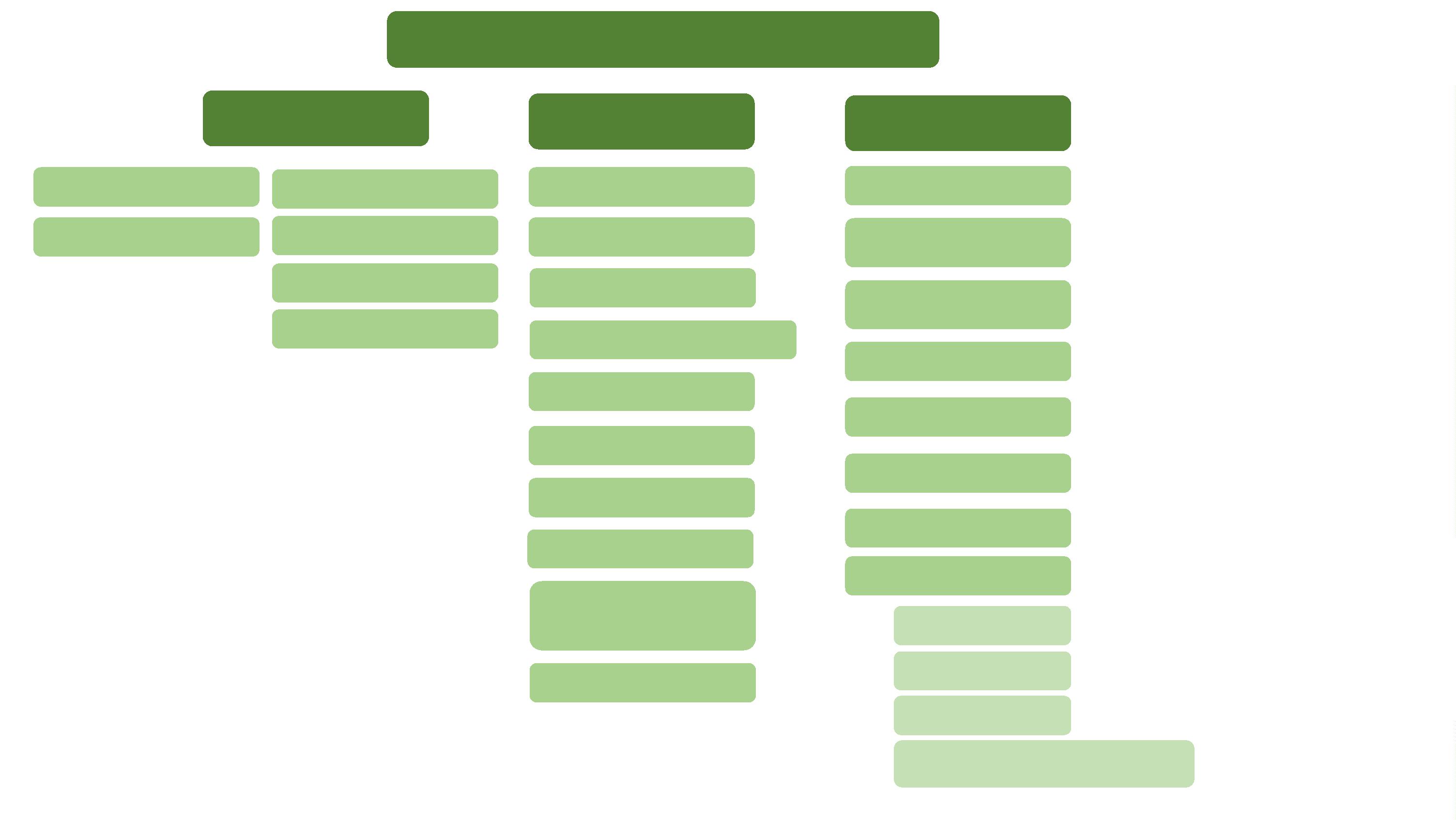


Special aspects

Patients

Doctors

little time

Hope for quick help

long ordeal

Cost factor

tired of explanations

Experience = inaccuracy

Uncertainties

Additional work by students

Relief through students

Desire "to be seen"

Excitement / tension

Personal contact request

Suffering pressure

Students

resource-saving

Equally experienced treatment

little experience = accuracy


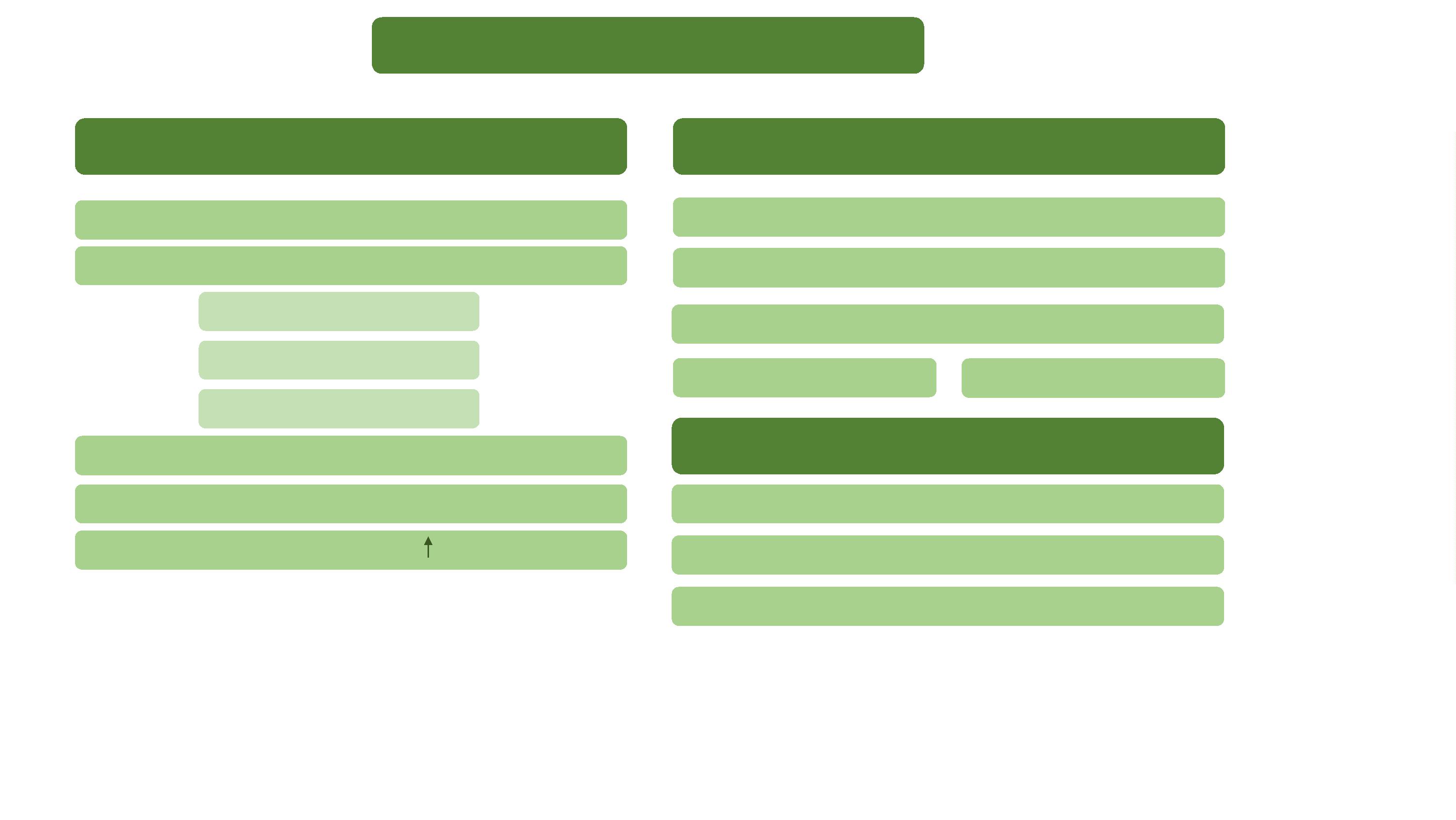


Prerequisites

Wishes & expectations

Standardised preparation

Accessibility

Student-led clinics

Good co-operation

Doctors & students

Contact persons

Accessibility

Short intervals between

appointments

Overall assessment by doctors

File/document

Advantages

Notes

General

Patients

Behavioural advice

for patients

Possible challenges

Relief for the clinic

Time saving

Medication

Openness for students

long ordeal

Practical training

Time saving

Intervention

Compensation for

staff shortages

Students

unbiased

Doctors

Reduced waiting

times

focussed work

Comprehensiveness

Relief


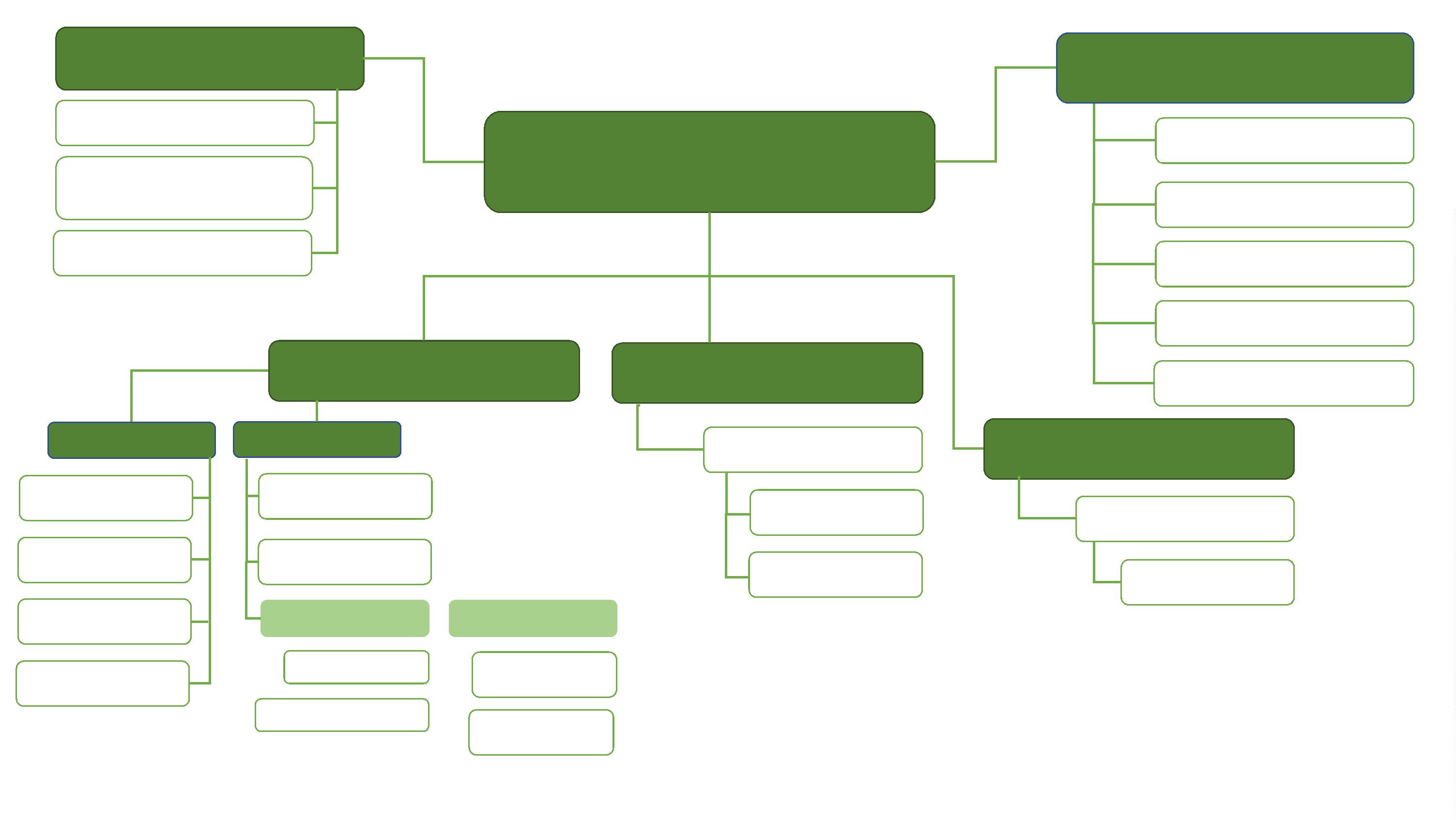

Supplement: Supplementary file 2 — Supplementary file2 (DOCX 4322 KB) [file 296_2023_5522_MOESM2_ESM.docx]
